# Embolie pulmonaire: la thrombolyse pourrait être élargie au risque intermédiaire?

**DOI:** 10.11604/pamj.2017.26.238.11172

**Published:** 2017-04-25

**Authors:** Abdelmajid Bouzerda, Issam Serghini, Abdenassar El Kharass

**Affiliations:** 1 Service de Cardiologie, 1 Centre Médico-Chirurgical, Université Cadi Ayyad, Faculté de Médecine et de Pharmacie de Marrakech, Maroc; 2 Service de Réanimation, 1 Centre Médico-Chirurgical, Agadir, Maroc; 3 Service de Radiologie, 1 Centre Médico-Chirurgical, Agadir, Maroc

**Keywords:** Embolie pulmonaire, risque intermédiaire, thrombolyse, Pulmonary embolism, intermediate risk, thrombolysis

## Abstract

La mortalité de l'embolie pulmonaire compliquée d'état de choc varie de 25 à plus de 50%. Les fibrinolytiques semblent donc justifiés dans cette situation, malgré la légère augmentation du risque hémorragique. Nous rapportons le cas d'une patiente admise pour une embolie pulmonaire à risque intermédiaire fort traitée avec succès par tenecteplase. A la lumière de cette observation et une revue de la littérature nous discutons l'indication des thrombolytique dans les embolies pulmonaire de gravité intermédiaire.

Mortality rates from pulmonary embolism complicated by shock vary from 25 to more than 50%. Therefore, fibrinolytic therapy appears to be justified in this situation, despite a slight increased risk of bleeding. We here report the case of a patient admitted with intermediate-risk pulmonary embolism successfully treated with tenecteplase. This study and review of the literature aim to discuss the indication for thrombolysis in patients with intermediate-risk pulmonary embolism.

## Introduction

L'embolie pulmonaire (EP) est une affection fréquente, grevée d'une morbidité et d'une mortalité importante. Depuis plus de trente ans, de nombreuses études ont évalué la place de la thrombolyse dans le traitement de l'EP grave. D'après des études récentes, les patients qui présentent une EP avec dysfonction ventriculaire droite pourraient bénéficier de ce traitement. Cet article a pour but de résumer les connaissances actuelles sur l'indication à la thrombolyse dans l'EP de gravité intermédiaire.

## Patient et observation

**Figure 1:**
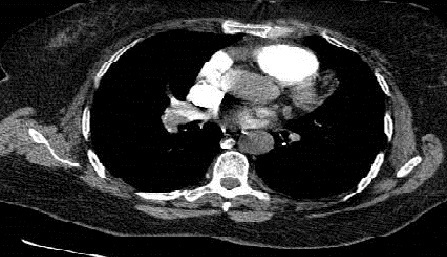
Angio-TDM montrant un énorme thrombus de l’artère pulmonaire droite

**Figure 2:**
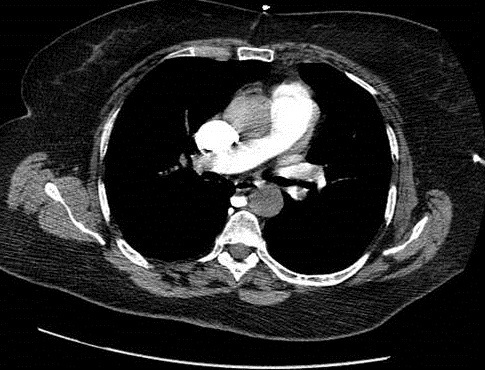
Angio-TDM montrant un thrombus de l’artère pulmonaire gauche

**Figure 3:**
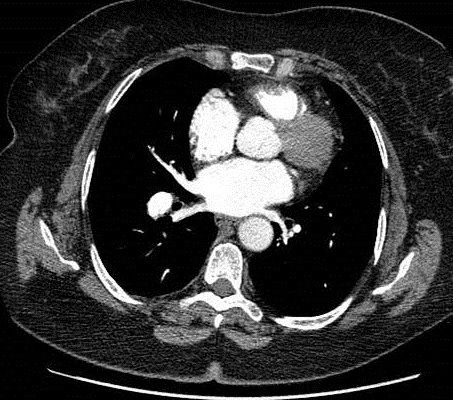
Contrôle à 6 mois montrant une disparition des thrombus

Mme H.M âgée de 48 ans consulte aux Urgences de l'hôpital pour une douleur thoracique survenant en postprandial et évoluant depuis 48 heures. Ses principaux antécédents sont une cure chirurgicale d'hernie discale datant de 20 jours avant son admission. On ne retrouvait pas de notion d'intoxication tabagique ni de voyage récent. L'histoire de la maladie débute par l'apparition brutale d'une douleur thoracique d'allure angineuse, sans notion de dyspnée ni palpitations, ni perte de connaissance, cette symptomatologie est associée à une fébricule. Après une amélioration spontanée, transitoire, la recrudescence des symptômes motive une consultation aux urgences. A l'admission, la pression artérielle est à 140/85 mm Hg, la fréquence cardiaque à 147/min, la SaO_2_ à l'air ambiant à 96%. L'auscultation cardiopulmonaire est normale en dehors de la tachycardie. L'examen physique ne met pas en évidence des signes d'insuffisance cardiaque. Les mollets sont souples et indolores et le reste de l'examen somatique est sans particularité. L'électrocardiogramme montre un rythme régulier sinusal à 140bt/min, avec un aspect de bloc de blanche droit incomplet et des extrasystolies ventriculaires isolées. La radiographie thoracique est sans anomalie. Les données complémentaires fournies par le bilan biologique sont une créatinine à 8,20 mg/l, une Hémoglobine à 14,2 g/dl, une élévation de la troponine hypersensible à 529 pg/ml, une augmentation du BNP à 1020 ng/L et une CRP à 7.90 mg/L. L'échocardiographie transthoracique montre une dilatation des cavités droites avec une hypocinésie de la paroi libre du ventricule droit, une cinétique paradoxale du septum interventriculaire, Un ventricule gauche non dilaté et non hypertrophié de fonction systolique conservée et il n'y a pas de valvulopathie mitroaortique. L'angioscanner thoracique réalisé en urgence montre un aspect en faveur d'une embolie pulmonaire bilatérale et extensive prédominant à droite (Figure 1, Figure 2). A ce stade, le diagnostique retenu est celui d'une embolie pulmonaire de gravité intermédiaire chez une femme jeune. Une anticoagulation efficace est immédiatement débutée par héparine non fractionnée par voie intraveineuse au pousse seringue. Le contrôle des paramètres hémodynamique note une tendance à l'hypoxémie sans véritable choc. Une décision de thrombolyse intraveineuse par Ténéctéplase est donc prise. L'évolution secondaire est favorable (diminution de l'oxygénodépendance, de la troponine et du taux de BNP). La tolérance du traitement thrombolytique est bonne, sans signe d'hémorragie. À 36 heures de la thrombolyse, la dysfonction ventriculaire droite a régressé sur l'échocardiographie de contrôle. L'angioscanner pulmonaire réalisé à 6mois montre une disparition totale des thrombus artériels pulmonaires (Figure 3).

## Discussion

L'embolie pulmonaire constitue une pathologie dont la symptomatologie est classiquement peu spécifique, rendant son diagnostic très difficile. La létalité hospitalière de l'embolie pulmonaire (EP) dépend essentiellement de l'existence de comorbidités et de la tolérance hémodynamique clinique [1, 2]. De l'ordre de 25 à 35% en présence d'un état de choc [1, 2], elle est inférieure à 5% en l'absence d'altération hémodynamique clinique et de comorbidité majeure [3]. Si le scanner spiralé est l'examen diagnostique de référence pour l'embolie pulmonaire, il est admis qu'il est souvent difficile à effectuer en cas d'instabilité hémodynamique. Dans ce cas, l'échocardiographie est considérée comme l'examen de référence [2]. Plusieurs signes échographiques permettent de faire le diagnostic de cœur pulmonaire aigu: dilatation du ventricule droit, cinétique septale paradoxale, hypertension artérielle pulmonaire, diminution du collapsus inspiratoire de la veine cave [4] ou encore hypocinésie du segment apical du ventricule droit [4]. L'élévation des biomarqueurs (troponine, peptide natriurétique B) et la présence d'anomalies morphologiques sur l'Echocardiographie doppler transthoracique (dilatation des cavités droites) témoignant de la dysfonction ventriculaire droite a permis de définir. L'embolie pulmonaire de gravité intermédiaire chez notre patiente. La gazométrie sanguine n'a pas été effectuée, elle aurait pu affiner le diagnostic mais n'aurait pas permis d'affirmer ou d'éliminer le diagnostic. Chez cette patiente, au pronostic vital engagé à très court terme, la décision d'administrer un thrombolytique a été prise après une réflexion du rapport bénéfice/risque. Nous avons utilisé la ténectéplase, seul thrombolytique disponible dans l'hôpital. S'il existe un consensus relativement large pour réaliser une fibrinolyse chez les patients présentant une EP grave, la question de la fibrinolyse des formes de gravité intermédiaire a longtemps été incertaine. Des résultats récents indiquent que la fibrinolyse réduit le risque de décompensation hémodynamique et de mortalité liée à l'EP mais est également associée à une augmentation significative des hémorragies graves, si bien que son utilisation doit être mûrement réfléchie chez de tels malades [5]. En fait la présence d'une dysfonction ventriculaire droite échographique ou d'une élévation des biomarqueurs étant responsable d'une augmentation de la mortalité précoce. Une première étude randomisée, conduite chez des patients avec EP et dysfonction ventriculaire droite échographique, montrait que l'altéplase était associée à une réduction des dégradations hémodynamiques sans incidence sur la mortalité. L'administration d'un bolus IV de tenectéplase en plus d'un traitement anticoagulant par HBPM a permis de réduire le taux d'évènements graves et d'améliorer la capacité fonctionnelle et la qualité de vie à 3 mois comparé au groupe de patients traités par HBPM seule dans l'étude TOPCOAT [6]. Dans l'étude PEITHO [5] qui a inclus 1006 patients avec EP à risque intermédiaire élevé (défini par une dysfonction ventriculaire droite et une élévation de la troponine), l'administration de tenectéplase était associée à une réduction de 56% du critère combiné décès ou détérioration hémodynamique à 7 jours (p = 0,015). Ce bénéfice, essentiellement lié à une réduction des épisodes de détériorations hémodynamiques (1,6% vs 5%; p = 0,002), était grevé par une majoration significative du risque d'hémorragies sévères (6,3% vs 1,5%; p < 0,001) et du risque d'hémorragies cérébrales (2% vs 0,2%). Les résultats de l'étude PEITHO ont conduit les auteurs des recommandations européennes à considérer que les patients presentant embolies à risque intermédiaire nécessitent une surveillance « armée » en unité de soins continus afin de permettre une stratégie de reperfusion (thrombolyse de sauvetage) en cas de dégradation hémodynamique. Cette utilisation de la fibrinolyse dans l'embolie pulmonaire à risque intermédiaire ne se conçoit donc que chez les malades de moins de 75 ans en l'absence de risque hémorragique. L'utilisation de la fibrinolyse in situ associée à des ultrasons serait à moindre risque hémorragique que la fibrinolyse classique et pourrait Constituer un traitement complémentaire intéressant en cas d'EP grave ou intermédiaire élevée à risque hémorragique [7].

## Conclusion

L'étude PEITHO ouvre des perspectives pour améliorer la prise en charge des EP à risque intermédiaire mais il semble que le traitement fibrinolytique ne se conçoit que chez les patients jeunes et en l'absence de risque hémorragique. Cette suggestion ne repose toutefois que sur des arguments indirects et de petites études ; elle demande à être mieux argumentée par des essais de plus grande ampleur.

## References

[1] Goldhaber SZ, Visani L, De Rosa M (1999). Acute pulmonary embolism: clinical outcomes in the International Cooperative Pulmonary Embolism Registry (ICOPER). Lancet..

[2] Kasper W, Konstantinides S, Geibel A (1997). Management strategies and determinants of outcome in acute major pulmonary embolism: results of a multicenter registry. J Am Coll Cardiol..

[3] Buller HR, Prins MH, Lensin AW (2012). Oral rivaroxaban for the treatment of symptomatic pulmonary embolism. N Engl J Med..

[4] Planquette B, Belmont L, Guy Meyer, Olivier Sanchez (2011). Prise en charge diagnostique et thérapeutique de l'embolie pulmonaire grave. Rev Mal Respir..

[5] Meyer G, Sanchez O, Planquette B (2015). Embolie pulmonaire de gravité intermédiaire: thrombolyse ou non?. Réanimation. Mars.

[6] Meyer G, Vicaut E, Danays T, Agnelli G (2014). Fibrinolysis for patients with intermediate-risk pulmonary embolism. N Engl J Med..

[7] Kucher N, Boekstegers P, Muller OJ, Kupatt C, Beyer-Westendorf J, Heitzer T (2014). Randomized controlled trial of ultrasound-assisted catheter-directed thrombolysis for acute intermediate-risk pulmonary embolism. Circulation..

